# Reply to Same

**Published:** 1883-10

**Authors:** E. J. Dunning


A REPLY BY E. J. DUNNING.

"Give us a Rest" is an earnest and agonizing cry, in which most of us heartily join. My friend, Dr. Blackham, in a very sprightly and well-written article, bemoans the seemingly endless and tiresome worship of Thomas Carlyle, and he belabors the kneeling devotees at this shrine with a vigor and heartiness that one must admire. It reminds me of that remote period in the world's history, before meat was cooked or roasted, when the responsible householder, Ho-Ti, returning from a journey or other absence, finds his poor dwelling in ashes, and that graceless whelp, Bo Bo, tearing the flesh from one of his nine precious young pigs that had perished in the flames, utterly absorbed in his horrid and unprecedented feast. The father showers blows upon his unnatural son, but can produce no impression except "O father, f ther! See how good the burnt pig eats!" The readers of the delightful Elia will remember that Bo-Bo, despite the parental cudgel, though the first, was not the only victim of the new fetich. Ho-Ti himself soon yielded to the enchantment, and even the jury before whom the scandalized neighbors dragged the bedeviled Ho-Ti and his Caliban of a brat, themselves became devotees; the stern judge finally yielded to the delights of the new religion; yea, the contagion affected the whole province, which was all ablaze with a perpetual holacaust. Insurance companies were wrecked, and their chop-fallen presidents united in a protest against the popular craze, at the head of which blazed the legend, "Give us a Rest!"

With sorrow, I feel bound in candor to say I fear the doctor's wearied and exasperated soul must still wait long years for the rest he so earnestly invokes. Our popular magazines have a thrifty way of filling their pages with such articles as their readers call for and find attractive. When the world ceases to love and honor the man who has, more than any other, moulded the thought and roused and stimulated the aspirations and the efforts of the men and women who have grown into maturity in England and America during the last fifty years, then the magazines and the newspapers will cease to write of him. When the many thousands of our English-speaking race, who thank "this objectionable Scotchman" for the impetus which, like a wave, has borne their lives to higher levels of thought and achievement than could have otherwise been reached, cease to delight in his books and to long for more and more knowledge of his personality, *then* books will cease to be written about him. If the friends of Mr. Carlyle do admit as many of the charges against him as the doctor recapitulates, and still remain his friends and worshippers, how great must be the love and reverence they feel for this greatest thinker of the age;—to whom the world owes so much, not only for his philosophy, but for his wonderful biographies and delineations of character, and for his unequalled histories?

The doctor says, "that Carlyle was disagreeable, obstinate, hot-tempered and selfish, his admirers all admit." Such sweeping generalities will apply to most men, I suppose. Many of the very best are sometimes, to some people, disagreeable, hot-tempered. etc., but that these qualities belong, in any special or peculiar way, to Thomas Carlyle, I, as one of his most insignificant admirers, do *not* admit. That Carlyle was, probably, not a perfect husband, and that Mrs. Carlyle was not a perfect wife, and that marriage to them brought its usual amount of discipline, through mutual, conscious and unconscious irritations. every reader of the four volumes containing the letters of both, published by Mr. Froude knows. But that either of them ever ceased, so long as life lasted, to feel for the other a deep, faithful and trusting love, no *attentive* and *sympathetic* reader can believe. Space will not permit me to follow the doctor *in extenso;* suffice it to say, most of the readers, admirers and friends of Mr. Carlyle, who have lived upon his books during his life, rejoiced in, and been elevated by his thought, mourned at his death and read his biography, love and honor him more, rather than less, for the revelations which this calcium light of Froude's volumes has cast upon the life, personality and relations of their great prophet. How unfortunate the doctor's criticism is, in one instance, will be seen in the following extracts from Froude's biography, which throws light upon one of his sentences, which is the last that I can notice: "A constant prater about honesty and reform, he was himself a seeker for an office for which he was not qualified, and he abused his friend Jeffrey in a way that would have filled the heart of a Billingsgate fish-woman with envy, when Jeffrey refused to use his influence to get him (Carlyle) appointed to the position of astronomer in an observatory." The following on this subject, by Mr. Froude, occurs on page 228, 2d vol. of Froude's Life of Carlyle:

"He was justly conscious of his qualifications. The mathematical ability which he had shown in earlier times, had been so remarkable as to have drawn the attention of Legendre. Though by the high standard by which he habitually tried himself, Carlyle could speak, and did speak, of his own capabilities, with mere contempt; yet he was above the affectation of pretending to believe that any really fitter candidate was likely to offer himself." Carlyle says in his diary, "I will this day write to Jeffrey about it. Any hope? Little. My care for it also not much. *Let us do what we can.* The issue not with *us.*" In a letter to his brother John, Carlyle writes, after his refusal by Jeffrey: "I have written back to the poor body, suppressing all indignation, if there were any; diffusing over all the balm of pity, and so in a handsome manner terminate the business. One has ever and anon a kind of desire to 'wash away' this correspondent of ours; yet really it was not right. I can see him even in this letter to be very thoroughly miserable, and am bound to help him, not aggravate him. His censures, too, have something flattering even in their violence—otherwise impertinent enough. He cannot tolerate me, but also he cannot despise me; and that is the sole misery. On the whole, dear Jack, I feel it very wholesome to have my vanity humbled from time to time. Would it were rooted out forever and a day! My mother said, when I showed her the purport of the letter, 'He canna hinder thee of God's providence,' which also was a glorious truth."

